# Characterizations of Hamster Retina as a Model for Studies of Retinal Cholesterol Homeostasis

**DOI:** 10.3390/biology10101003

**Published:** 2021-10-06

**Authors:** Nicole El-Darzi, Natalia Mast, Brian Dailey, John Denker, Yong Li, Joseph Vance, Irina A. Pikuleva

**Affiliations:** 1 Department of Ophthalmology and Visual Sciences, Case Western Reserve University, Cleveland, OH 44106, USA; nae20@case.edu (N.E.-D.); nvm2@case.edu (N.M.); bxd238@case.edu (B.D.); jad6@case.edu (J.D.); yxl665@case.edu (Y.L.); 2 Spective LLC, Durham, NC 27705, USA; jvance@thespectivegroup.com

**Keywords:** hamster, retina, cholesterol, retinal abnormalities, retinal blood vessels, diabetes, diabetic retinopathy

## Abstract

**Simple Summary:**

This work represents a comprehensive evaluation of hamster retina by state-of-the-art methodologies and provides evidence that hamsters may represent a better model for studies of retinal cholesterol maintenance than mice. The latter is an important finding, as disturbances in retinal cholesterol homeostasis are linked to age-related macular degeneration and diabetic retinopathy, which are blinding diseases.

**Abstract:**

Cholesterol homeostasis in the retina, a sensory organ in the back of the eye, has been studied in mice but not hamsters, despite the latter being more similar to humans than mice with respect to their whole-body cholesterol maintenance. The goal of this study was to begin to assess hamster retina and conduct initial interspecies comparisons. First, young (3-month old) and mature (6-month old) Syrian (golden) hamsters were compared with 3- and 6-month old mice for ocular biometrics and retinal appearance on optical coherence tomography and fluorescein angiography. Of the 30 evaluated hamsters, seven had retinal structural abnormalities and all had increased permeability of retinal blood vessels. However, hamsters did not carry the mutations causing retinal degenerations 1 and 8, had normal blood glucose levels, and only slightly elevated hemoglobin A1c content. Cholesterol and six other sterols were quantified in hamster retina and compared with sterol profiles in mouse and human retina. These comparisons suggested that cholesterol turnover is much higher in younger than mature hamster retina, and that mature hamster and human retinas share similarities in the ratios of cholesterol metabolites to cholesterol. This study supports further investigations of cholesterol maintenance in hamster retina.

## 1. Introduction

Among rodents, mice and rats are perhaps the most common laboratory animals due to their small size, rapid breeding, low maintenance cost, and ease of genetic manipulations. Hamsters are a less utilized model, even though they are also small and belong to the same *Rodentia* order as mice and rats. Hamsters diverged from mice and rats at the family level and represent the rodent family *Cricetidae*, whereas mice are from the *Muridae* family [1]. There are 19 species in the *Cricetidae* family, with the most used laboratory models being Syrian (golden) hamsters (*Mesocricetus auratus*), Chinese (dwarf) hamsters (*Cricetulus griseus*), and Djungarian (or Siberian) hamsters (*Phodopus sungorus*) [1]. Yet none of these models have been characterized for cholesterol homeostasis in the retina, a sensory organ lining the back of the eye. This is in contrast to mice, whose retinal cholesterol maintenance has been intensively studied [2,3] but has not yet been compared to that of hamsters, which are more similar to humans than mice in many aspects of whole-body cholesterol maintenance [4].

Indeed, unlike mice, hamsters and humans express cholesterol ester transfer protein and hence have significant amounts of cholesterol in their systemic circulation on low density lipoprotein (LDL) particles, which supply cholesterol to many organs. Mice lack cholesterol ester transfer proteins and therefore carry most of their plasma cholesterol on high density lipoprotein (HDL) particles, which deliver excess cholesterol back from extrahepatic organs to the liver for further degradation to bile acids [5,6,7]. Another difference is that mice have both hepatic and intestinal editing of the gene for apolipoprotein B (*Apob*) and therefore mainly produce the APOB48 isoform of this apolipoprotein. In contrast, hamsters and humans have only intestinal *Apob* editing and thus express both apolipoprotein isoforms, APOB100 and APOB48, with the latter lacking the LDL receptor-binding domain [8]. Accordingly, the particles, which contain APOB100 (LDL), are taken up by cells via the LDL receptor-mediated endocytosis, whereas the particles, which contain APOB48 (chylomicrons), are processed via a different mechanism, which clears them from the systemic circulation faster than the APOB100-containing LDL [9,10]. Lastly, mice tolerate a dietary cholesterol challenge much better than humans and hamsters and thereby are more resistant to diet-induced atherosclerosis. This is in part because mice, hamsters, and humans have very different rates of whole-body cholesterol biosynthesis (160, 40, and 10 mg/day/kg, respectively) and different contributions by the liver to this rate (40%, 35% and 10%, respectively) [11]. As a result, when dietary cholesterol is present and suppresses hepatic cholesterol biosynthesis, mice can compensate more for the absorbed cholesterol than hamsters and humans. Moreover, mice remove dietary cholesterol by upregulating their bile acid biosynthesis, a mechanism that is absent in hamsters and humans as in both species bile acid production is not sensitive to dietary cholesterol [12,13,14].

In mouse retina, cholesterol is known to originate from two sources—in situ biosynthesis and tissue uptake of cholesterol-containing lipoprotein particles from the systemic circulation; the latter is mediated by the receptors for LDL and HDL [15,16,17,18,19,20,21]. Cholesterol biosynthesis is the major source of retinal cholesterol in mice and accounts for 72% of total retinal cholesterol input [22]. Consequently, retinal uptake of the blood cholesterol contributes 28% of the total retinal cholesterol input in mice. Yet it is not clear whether this uptake depends on the plasma ratio of LDL/HDL and would be higher in species like humans and hamsters, which have a higher plasma LDL content than mice. This knowledge is important as it provides insight into how retinal cholesterol homeostasis is maintained in mammals and what species represent better models for humans. The latter is critical for developing therapeutic strategies to treat age-related macular degeneration and diabetic retinopathy, as disturbances in retinal cholesterol maintenance have been linked to these blinding diseases [23,24,25,26]. However, studies on hamster retina are still scarce and lack comprehensive retinal characterization by different imaging modalities and state-of-the art techniques. The present study fills this gap in our knowledge and provides some unexpected findings.

## 2. Materials and Methods

### 2.1. Animals

Female and male Syrian hamsters (Charles River, strain code: 049) were purchased at 9 weeks of age and housed with one animal per cage until the age of 3 or 6 months. Female and male C57BL/6J mice (The Jackson Laboratory, stock No: 000664) were purchased at the age of 8 weeks and housed five animals per cage until 3 and 6 months of age. Animals were maintained on a standard 12 h light (approximately 10 lux) dark cycle and were fed standard rodent chow; water was provided ad libitum. All animal procedures were approved by the Case Western Reserve University (Cleveland, OH, USA) Institutional Animal Care and Use Committee and conformed to the guidelines of the United States Department of Agriculture, the American Veterinary Association Panel on Euthanasia, and the Association for Research in Vision and Ophthalmology.

### 2.2. Study Design

Hamsters reach maturity at 6 months of age, whereas mice mature at 3 months of age [27,28]. Six-month-old hamsters (6 females, F1-F6, and 6 males, M1-M6) were characterized first (Table S1) and compared to mice of the same age in several cases. Hamster characterizations included genotyping, in vivo imaging of the retina, ocular biometric assessments, measurements of the levels of blood glucose, blood hemoglobin A1c, serum lipids, and retinal sterols along with retinal histological analysis to correlate with retinal abnormalities that were detected in some of the hamsters by in vivo imaging. We then decided to ascertain how frequent the retinal abnormalities are, and whether they are present in younger animals. A second group of 18 hamsters (9 females, F7-15, and 9 males, M7-M15) was purchased, genotyped, and aged to 3 months, upon which most were evaluated by retinal in vivo imaging and measurements for both blood glucose and HbA1c. Next, to quantify retinal sterols and carry out retinal histo- and immunohistochemistry, six female (F8, F10, and F12-F15) and six male hamsters (M10-M15) were euthanized, and the remaining three animals of each sex (F7, F9, F11, and M7-M9) were grown until the age of 6 months. These hamsters were then characterized by in vivo imaging of the retina as well as their measurements of blood glucose and HbA1c levels, serum lipids, and retinal sterols. Thus, a total of 30 hamsters were characterized: six female and six male hamsters at 3 months of age; three female and three male hamsters at both 3 and 6 months of age; and six female and six male hamsters at 6 months of age.

### 2.3. Anesthesia

Prior to all experiments, hamsters were anesthetized via intraperitoneal injections of 160 mg/kg ketamine and 7.5 mg/kg xylazine (Patterson Veterinary, Greeley, CO, USA, 07-890-8598 and 07-808-1947, respectively). Animals were kept warm on heating pads and remained surgically sedated throughout the experiment. Subcutaneous injections of aqueous atipamezole, 0.75 mg/kg (Patterson Veterinary, 07-867-7097), were used to wake up the animals.

### 2.4. Genotyping

Mouse and hamster genomic DNAs were sequenced for the mutations that lead to retinal degenerations 1 and 8 (Rd1 and Rd8, respectively). These are the naturally occurring nucleotide deletion in exon 9 of the *Crb1* (crumbs homologue 1) gene and the nonsense mutation in exon 7 of the *Pde6β* (phosphodiesterase 6β) gene. Genomic DNAs were obtained either from whole blood or ear tissue by using DNeasy Blood and Tissue kit (Qiagen, #69504) or DNA homogenization buffer (100 mM Tris-HCl, pH 8.3, containing 250 mM KCl, 0.1 mg/mL gelatin, 0.45% NP-40, 0.45%Tween 20, and 10 mg/mL proteinase K), respectively. To genotype for Rd8, DNAs were initially sequenced with the same primers that were used for sequencing mouse Rd8: the forward primer was 5′-CTGTCTGAGCACAATAGAGATTGG-3′ and the reverse primer was 5′-GGTGTATCCAGGCTCACAC-3′. The PCR conditions were as follows: initial denaturation was at 94° for 2 min followed by 41 cycles of 94° for 15 s, 60° for 30 s, and 72° for 15 s. The PCR products obtained were processed with the Qiagen PCR purification kit (Qiagen, Germantown, MD, USA, #28106) and sequenced with the forward primer. To confirm the sequencing data, the hamster-specific primers were then generated (the 5′-CAGACATCTATGTAGGTGACCAAG-3′ forward primer and the 5′-CACAGACAA GAATAGCCATGACTTC-3′ reverse primer) and used for PCR. The PCR products were processed and resequenced with the hamster-specific forward primer.

To genotype for Rd1, which in addition to the nonsense mutation in *Pde6β* is also associated with the xenotropic murine leukemia virus 28 (XMV) insertion in intron 1 of the same gene, the region of the *Pde6β* nonsense mutation was amplified with the hamster-specific primers (the forward 5′-CTCAGAGAAGTGAATAAAGTAAACTCATGG-3′ primer and reverse 5′-CCTTGCCTACAGGACAGGAG-3′ primer). The PCR conditions were as follows: initial denaturation was at 94° for 2 min followed by 37 cycles of 94° for 15 s, 57° for 30 s, and 72° for 15 s. The PCR products obtained were processed with the Qiagen PCR purification kit and sequenced with the forward primer. To check for the presence of the XMV insertion, primers specific to the non-repeated region of XMV were initially used: forward 5′-CACGTGATTCTACTTCTTCTGGATC-3′ and reverse 5′-GTCTCTGAC CTCGTTGTCTGAG-3′. The PCR conditions were as follows: initial denaturation was at 94° for 2 min followed by 37 cycles of 94° for 15 s, 57° for 30 s, and 72° for 15 s. Then, since this insertion was present in many of the samples, the hamster-specific forward primer (the 5′-CATGCCTACCCATGTGCTAC-3′) upstream of the XMV insertion in mouse *Pde6β* and the XMV-specific reverse primers (the 5′-AAGCTAGCTGCAGTAACGCCATTT-3′) or (the 5′-CCTTGCCTACAGGACAGGAG-3′) were used to link the XMV insertion to *Pde6β*. The PCR conditions were as follows: initial denaturation was at 94° for 2 min followed by 37 cycles of 94° for 15 s, 57° for 30 s, and 72° for 15 s.

### 2.5. In Vivo Imaging

Retinal imaging by ultra-high resolution spectral-domain optical coherence tomography (SD-OCT) and fluorescein angiography (FA) were carried out as described in [29,30]. An Envisu R2200 UHR OCT imaging system (Leica Bioptigen, Morrisville, NC, USA) with a custom-made stage provided by Leica Bioptigen and a scanning laser ophthalmoscope (Spectralis HRA + OCT, Heidelberg Engineering, Franklin, MA, USA) were used, respectively, for each imaging modality. Images for FA were acquired after a bolus (0.1 mL) intraperitoneal injection of 5.0% sodium fluorescein (Akorn Inc, Lake Forest, IL, USA, #17478-250-20) in phosphate-buffered saline (PBS). Fundus imaging (FI) was carried out using Ivivo fundoscope (Xenotec OcuScience, Henderson, NV, USA) according to the manufacturer’s instructions.

### 2.6. The Axial Eye Length Measurements by SD-OCT

The maximum image depth for the SD-OCT instrument that was used is limited to 1.6 mm in-tissue. Hence, the fixed-focus Leica M50 Mouse Retina lens can image the hamster retina and cornea through the adjustment of the working distance (i.e., the distance from the OCT lens to the corneal surface) and the reference arm position. To adjust the working distance, it was set to focus midway along the optical axis such that the retina and cornea were resolved sufficiently to mark each surface accurately, using the on-screen calipers provided by the InVivoVue software. Then, the reference arm position was adjusted first to bring the anterior cornea surface to an on-screen fiducial (horizontal line at fixed axial location) and then to bring the choroid to the same fiducial, leaving the working distance fixed. Both reference arm positions were recorded and entered into the following equation to estimate the axial length to within the accuracy of the default Refractive Index (1.38): Axial Distance = [Reference Arm Position Count (RPE Layer) − Reference Arm Position Count (Anterior Cornea Surface)] × [(0.127mm/count)/(1.38)].

### 2.7. Blood Glucose

The cheek pouches of hamsters were emptied with rubber-tipped forceps, their bedding was removed, and animals were fasted overnight for 12 h. The next morning, a drop of blood was collected from the lateral saphenous vein and was assayed for glucose by an Elite XL Glucometer (Bayer Contour, Parsippany, NJ, USA).

### 2.8. Euthanasia

Animals were either non-fasted or fasted overnight for 12 h as described above and then deeply sedated in the morning with a 200 mg/kg ketamine and 10 mg/kg xylazine bolus injection. Blood was withdrawn via cardiac puncture, animals were decapitated by guillotine, and eyes were enucleated.

### 2.9. Blood Hemoglobin A1c (HbA1c)

Fasted or non-fasted blood was withdrawn either from the saphenous vein or via cardiac puncture and was placed in eppendorf tubes coated with 0.5 M aqueous EDTA. An aliquot of 20 µL was then taken to measure the HbA1c content by a kit from Crystal Chem, Inc. (Elk Grove Village, IL, USA, #80310) according to the manufacturer’s instructions.

### 2.10. Serum Lipids

Serum was isolated from fasted blood as described in [31] and was sent to the IDEXX Laboratories, Inc. (Westbrook, ME, USA) for measurements of total, HDL, and LDL cholesterol and triglycerides.

### 2.11. Sterol Profiling

Individual retinas were used. Retinal isolation, processing, and sterol profiling were carried out as described in [30,32]. Sterol quantification was conducted by isotope dilution gas chromatography-mass spectroscopy using deuterated sterol analogs as internal standards [33]. The measurements were of unesterified and total cholesterol (the latter is a sum of esterified and unesterified cholesterol) and other unesterified sterols.

### 2.12. Histo- and Immunohistochemistry

The preparation of paraffin and plastic sections for stains with hematoxylin and eosin (H&E, Thermo Fisher Scientific, Inc., Waltham, MA, USA, #6765007 and # 6766007, respectively) and toluidine blue (Electron Microscopy Sciences, Hatfield, PA, USA, #22050), and of frozen sections with filipin (Cayman Chemical, Ann Arbor, MI, USA) were carried out as described in [21,29,30].

Staining of frozen sections for albumin and retinal blood vessels were conducted as described in [34]. To visualize albumin, rabbit anti-albumin antibody (Abcam, Waltham, MA, USA, ab207327) at a 1:500 dilution in PBS containing 5% normal goat serum (NGS, Life Technologies, Frederick, MD, USA, PCN5000) and 0.05% Tween 20 (Fisher Scientific, Fair Lawn, NJ, USA, #BP337-500) was used. To outline the blood vessels, sections were incubated with isolectin GS-IB4 conjugated to Alexa Fluor-594 (Invitrogen, Waltham, MA, USA, #121413, 1:100 dilution in PBS). Slides were imaged on an Olympus Fluoview FV1200 Laser Scanning Confocal Microscope. For BRN3A stains, retinal sections were subjected to heat-mediated (90 °C for 30 min) antigen retrieval with 10 mM Na citrate buffer, pH 6.0, containing 0.05% Tween 20. Sections were then cooled to room temperature and blocked/permeabilized with PBS containing 5% NGS and 0.1% Triton X-100. Sections were washed three times for 5 min with PBS and incubated overnight at 4 °C with the primary rabbit monoclonal antibody to BRN3A (Abcam, ab245230, diluted 1:200 with PBS containing 5% NGS and 0.1% Triton X-100). The next morning, sections were washed with PBS three times and incubated for 1 h at room temperature with the secondary antibody, which was goat anti-rabbit Alexa Fluor 647 (Jackson ImmunoResearch, West Grove, PA, USA, 111-605-144, diluted 1:200 with PBS containing 5% NGS and 0.1% Triton X-100). Then, sections were washed three times with PBS, covered with DAPI Fluoromount-G (SouthernBiotech, Birmingham, AL, USA, 0100-20), and protected with a glass coverslip. Slides were imaged on a Zeiss AxioScan.Z1 with the high performance Hamamatsu ORCA-Flash4.0 v3 monochrome camera, a 20x/0.8 Plan-Apochromat objective, and a Colibri 7 Illumination kit (Carl Zeiss Research Microscopy Solutions, White Plains, NY, USA).

### 2.13. Statistical Analysis

Data from all available animals were used. There was no exclusion of statistical outliers. Data represent the mean ± SD; the sample size (n) is indicated in each figure or figure legend and was based on previous experience. For data sets with *n* > 8, normality of distribution was assessed by the D’Agostino–Pearson test; when *n* < 8, the Shapiro–Wilk test was used. Unpaired non-parametric Mann–Whitney test was used when the data were not normally distributed. In all other cases, either a two-tailed, unpaired Student’s *t*-test or one-way ANOVA with Tukey’s multiple comparisons test was used. Statistical significance was defined as * *p* ≤ 0.05; ** *p* ≤ 0.01; *** *p* ≤ 0.001.

## 3. Results

### 3.1. Genotyping for the Mutations That Underlie Rd1 and Rd8

These mutations are frequent in mice [35]. Therefore, we investigated whether they are present in hamsters. Genotyping was used because Rd1 and Rd8 cannot be unambiguously detected by fundus examination if the underlying mutations are heterozygous. In addition, the manifestations of Rd8 vary strongly with genetic background; hence fundus examination alone may not be sufficient to identify this mutation. In the *Crb1* gene, none of the hamsters had a nucleotide deletion that underlies Rd8 (Figure S1). However, the 198-bp region encompassing Rd8 had a difference in 14 nucleotides, or 6 amino acid residues, between hamsters and mice. Notably, one nucleotide change was in the codon of Rd8 and led to the amino acid substitution from Arg in mice to Gln in hamsters (Figure S1). In the *Pde6β* gene, none of the hamsters contained the nonsense mutation that causes Rd1, yet one animal (M5) contained the XMV insertion in intron 1, which is associated with Rd1. Overall, the 67-bp region encompassing the Rd1-containing exon 7 had a difference in 2 nucleotides between hamsters and mice, which, however, did not lead to any changes in the amino acid sequence (Figure S2).

### 3.2. Ocular Biometric Assessments

**Figure 1:**
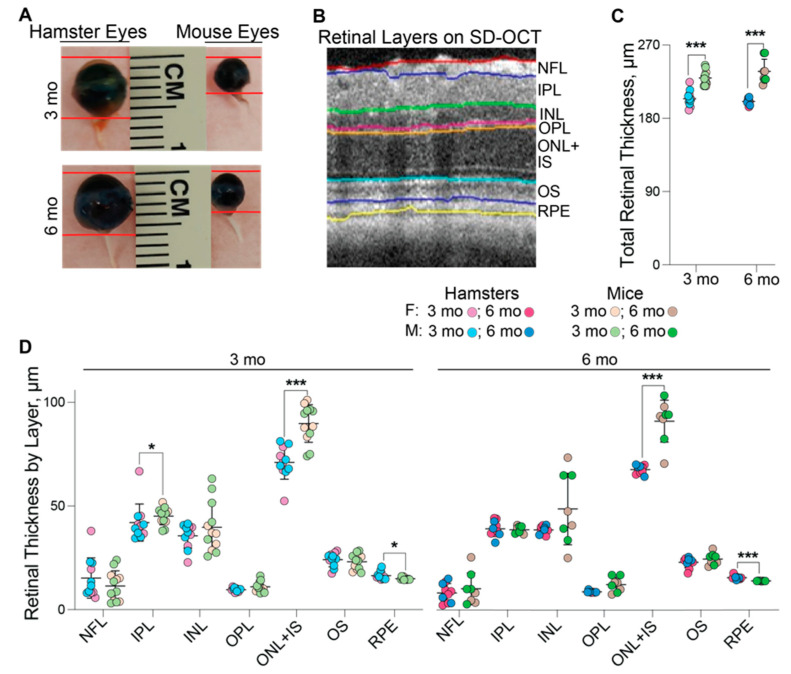
Comparative biometric assessments of hamster vs. mouse eyes and retina. (**A**): Representative images of hamster (3 females and 3 males) and mouse (4 females and 3 males) eyes at 3 months (3 mo) and 6 months (6 mo) of age. (**B**): The reference image of mouse retina provided by the Diver software to show how retinal layers in hamsters and mice were defined for quantifications by spectral-domain optical coherence tomography (SD-OCT). (**C**): Total retinal thickness by SD-OCT in 3-month-old hamsters (5 females and 6 males), 3-month-old mice (5 females and 7 males), 6-month-old hamsters (6 females and 4 males), and 6-month-old mice (4 females and 4 males). (**D**): Retinal thickness by layer in 3-month-old hamsters (5 females and 6 males), 3-month-old mice (5 females and 7 males), 6-month-old hamsters (6 females and 4 males), and 6-month-old mice (4 females and 4 males). No sex-based differences were detected, hence data from female and male animals were combined. Data represent the mean ± SD of the measurements in individual animals after the data from both eyes were averaged. Statistical significance was assessed by an unpaired non-parametric Mann–Whitney test. * *p* ≤ 0.05; ***, *p* ≤ 0.001. F, females; M, males; NFL, the nerve fiber layer; IPL, the inner plexiform layer; INL, the inner nuclear layer; OPL, the outer plexiform layer; ONL, the outer nuclear layer; IS, the photoreceptor inner segments; OS, the photoreceptor outer segments; RPE, the retinal pigment epithelium.

These assessments were comparative and in addition to hamsters included mice at 3 and 6 months of age, despite mice reaching maturity at 3 months of age. SD-OCT and a ruler were used to measure the eye axial length or the distance between the cornea and the optic nerve (Figure 1A, Table 1). Both approaches produced similar measurements and documented a lack of sex-based differences in animals of the same age and species. However, the advantage of the measurements by SD-OCT is that this approach could be done on live animals and does not require animal euthanasia. The axial eye length did not differ significantly between 3 and 6 months of age in either hamsters or mice, but the hamster eye length was almost two times greater than that in mice (~6 mm vs. ~3.3 mm). Accordingly, the wet weight of the retina at 3 and 6 months of age was 3.0- and 3.3-times greater in hamsters than in mice. Additionally, in 6-month-old hamsters and mice, the retina contained ~0.8 mg and ~0.25 mg of total protein, respectively (not shown). Nevertheless, despite these differences, hamster retina appeared to be slightly thinner than mouse retina as indicated by the quantifications of retinal cross sections by SD-OCT at the level of the optic nerve. The total retinal thickness in hamsters vs. mice was 204.2 µm vs. 229.4 µm at 3 months of age and 200.7 µm vs. 237.6 µm at 6 months of age with no differences between male and female animals (Figure 1B,C, Table 1). As compared to mice, hamsters had a decreased thickness of the layer encompassing the outer nuclear layer and the photoreceptor inner segments at both 3 and 6 months of age (71.0 and 70.3 µm vs. 90.0 and 94.5 µm, respectively) and the inner plexiform layer at 3 months of age (41.9 µm and 45.1 µm in hamsters and mice, respectively). Conversely, the retinal pigment epithelium was slightly thicker in hamsters than in mice (16.4 and 16.2 µm vs. 15.0 and 14.5 µm, respectively) at both ages (Figure 1D).

### 3.3. In Vivo Imaging of the Retina and Area Centralis

**Figure 2:**
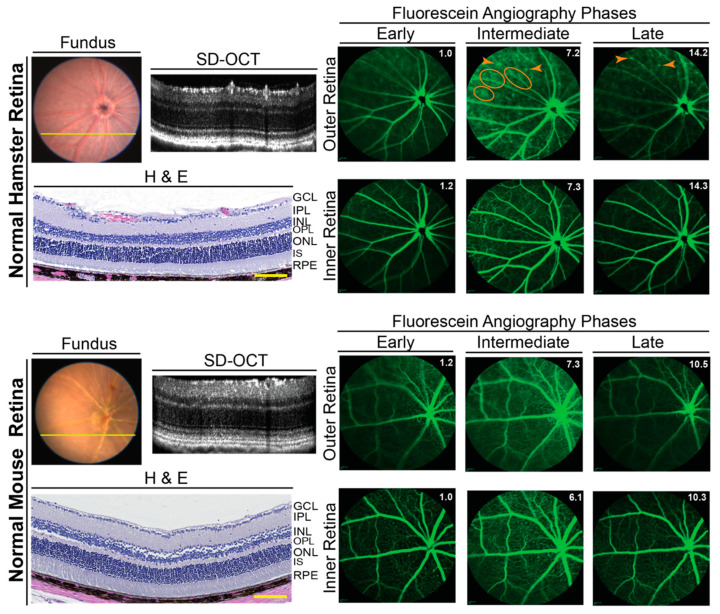
In vivo imaging of hamster and mouse retina. Representative (9 female and 9 male hamsters at 3 months of age; 9 female and 9 male hamsters at 6 months of age; 6 female and 6 male mice at 6 months of age) assessments by fundus color imaging (Fundus), spectral domain-optical coherence tomography (SD-OCT), and fundus fluorescein angiography after an injection with sodium fluorescein. No sex- and age-dependent differences were detected, hence only images of male animals are shown. The same representative animal (hamster or mouse) was used for the acquisition of all images. The SD-OCT panels shows retinal cross sections. Histology of the retinal cross sections after H&E stains is shown for comparison. The fluorescein angiography panels show an early-, intermediate-, and late-stage fundus fluorescence (from left to right) as defined by the post-injection time of image acquisition. This time (in minutes) is shown as white numbers in the upper right corner of each panel. The laser beam was focused on either the outer retina or inner retina, which are nourished by the choroidal and intraretinal vascular networks, respectively. Orange ovals and arrowheads on fluorescein angiography indicate diffused margins of some of the retinal capillaries and bright fluorescent spots, respectively. The retinal layer labeling is as described in Figure 1. GCL, the ganglion cell layer. Scale bars = 100 µm.

Three imaging modalities were used: SD-OCT to assess retinal gross structure; FA to visualize retinal blood vessels, and FI to complement SD-OCT and FA. All three suggested that, in general, hamster retina looked very similar to mouse retina, despite differences in length and width (Figure 2). Additional characterizations of retinal cross sections by light microscopy showed that histologically, the hamster and mouse retina were also similar (Figure 2).

**Figure 3:**
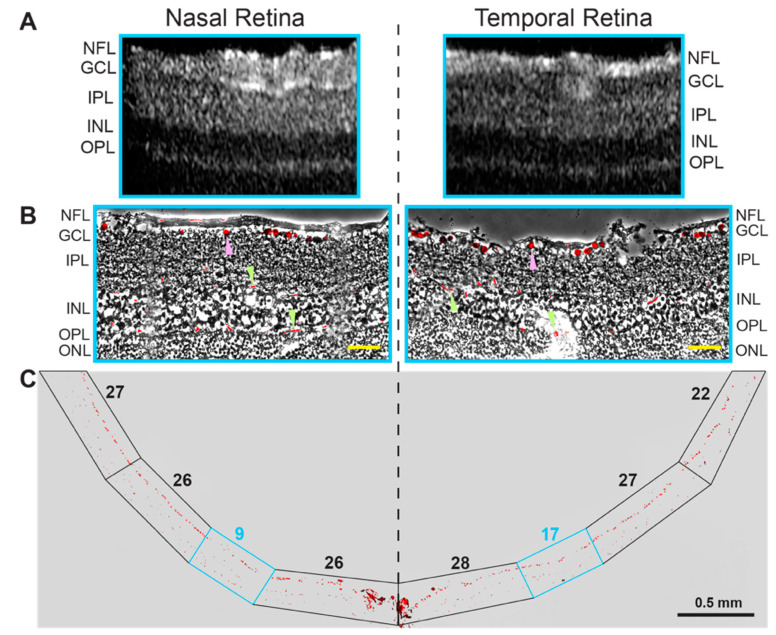
Localization of area centralis in hamster retina. Images of a representative retina (2 female and 3 male hamsters) were used, which was characterized by SD-OCT (**A**) and a stain for BRN3A (**B**,**C**). (**B**): An overlay of the BRN3A stain on the phase contrast image of the retina, which corresponds to the region in the cyan box in (**A**,**C**). Magenta arrowheads point to the BRN3A staining of the ganglion cells, whereas green arrowheads indicate staining of red blood cells inside the retinal blood vessels. (**C**): the BRN3A staining of the entire retinal cross section. Boxed regions indicate segments of the retina, in which the BRN3A-positive cells in the ganglion cell layer were counted and indicated by a number above the boxed region. The retinal layer labeling is as described in Figure 1 and Figure 2. Yellow scale bars = 50 µm.

Unlike mice, hamsters have an area centralis, a specialized region in the retina with a higher density of ganglion cells and a higher vision resolution than the rest of the retina [36]. This region is a prototype of the macula in humans responsible for sharp, clear, and colored vision. We surmised that the area centralis could be more hyperreflective on the B-scan of SD-OCT and used SD-OCT in an attempt to localize the area centralis 0.25–0.29 mm temporal and 0.03–0.09 mm superior to the optic nerve. This location was suggested previously based on ganglion cell counting in retinal flat mounts [36]. However, we could not obtain a conclusive result as exemplified by a comparison of retinal hyperreflectivity of the ganglion cell layers in the nasal and temporal retinal sides (Figure 3A). Hence, we used retinal cross sections in this region to stain for BRN3A, a transcription factor and nuclear marker of the retinal ganglion cells [37,38]. Indeed, counting of the BRN3A-positive cells in these sections identified a region with an increased cell count (Figure 3B,C), which could correspond to area centralis. However, we believe that the evidence obtained may not be sufficient for a definite conclusion; therefore, further studies using other approaches are required to unambiguously identify the area centralis in hamster retina.

### 3.4. Hamster Retinal Abnormalities

**Figure 4:**
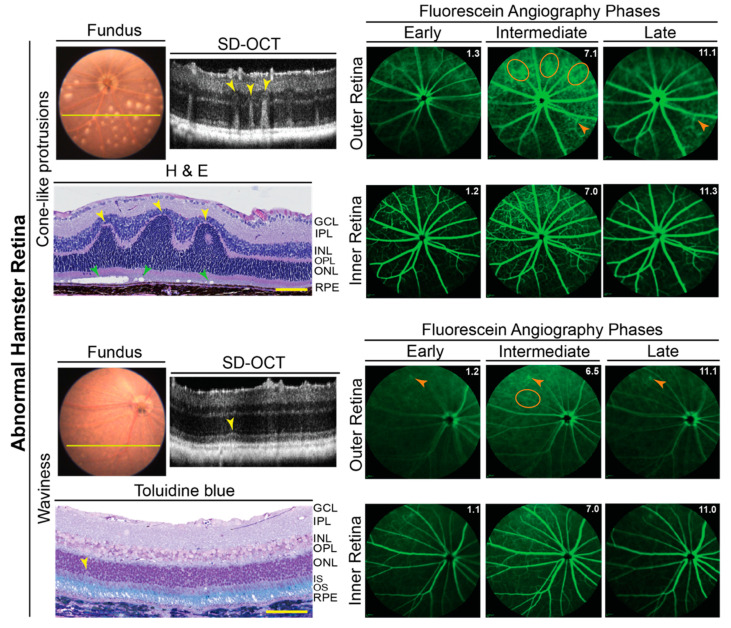
Abnormalities in hamster retina as assessed by in vivo imaging and histology. Representative images (two females and one male for cone-like protrusions; one female and three males for retinal waviness) are of fundus, retinal SD-OCT, fundus fluorescein angiography after an injection with sodium fluorescein, and histology with H&E and toluidine blue stains. For each pathology type (cone-like protrusions and retinal waviness), the same hamster was used for acquisition of all images. Yellow lines on fundus photos indicate location of abnormality seen on SD-OCT. Yellow arrowheads indicate structural abnormalities detected by both SD-OCT and histology. Green arrowheads indicate the degeneration of the photoreceptor outer segments revealed only by histology. Orange ovals and arrowheads indicate diffused margins of some of the retinal capillaries and bright fluorescent spots, respectively, on fluorescein angiography. For fluorescein angiography imaging, the laser beam was focused on either the outer retina or inner retina, which are nourished by the choroidal and intraretinal vascular networks, respectively. White numbers in the upper right corner of each fluorescein angiography panel indicate the post-injection time (in minutes) of image acquisition. The retinal layer labeling is as described in Figure 1 and Figure 2. Scale bars = 100 µm.

The B-scans of SD-OCT revealed that the retinas were structurally normal for only 23 of 30 evaluated hamsters or 77% of animals. In the remaining 7 hamsters, three animals (F10, M13, and F14) had cone-like protrusions from the outer to the inner retina, which corresponded to the whitish spots on FI (Figure 4). Four hamsters (M1, M2, F3, and M12) had occasional waviness of the ellipsoid zone of photoreceptors (the interface between the inner and outer photoreceptor segments [39]) and a parallel waviness of the overlaying external limiting membrane (Figure 4). However, no whitish spots were observed on FI.

Retinal lesions detected by SD-OCT were correlated to histology. Paraffin or plastic sections were obtained in precise alignment with the OCT images and stained with H&E or toluidine blue to confirm retinal structural abnormality. The cone-like protrusions on SD-OCT corresponded to occasional retinal folds, involving the outer and inner nuclear layers (Figure 4). There was also a degeneration of the photoreceptor outer segments under these folds as indicated by empty spaces. Occasional waviness of the ellipsoid zone of the photoreceptors on SD-OCT corresponded to a loss of nuclei in the outer nuclear layer, which is formed by the photoreceptor cell somas. Overall, both the cone-like protrusions and waviness were similar to the manifestations of Rd8 reported previously in mice [40].

**Figure 5:**
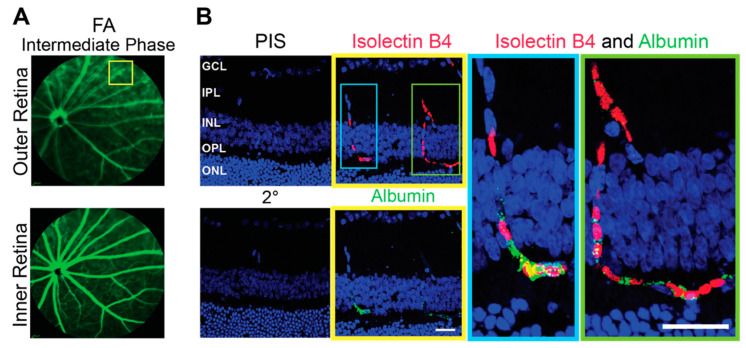
Increased permeability of the retinal blood vessels in hamsters. (**A**): Representative image (4 females and 3 males either with structurally normal or abnormal retinas, the staining pattern was the same) of a fundus fluorescein angiography (FA) showing an area (yellow box) of increased vasopermeability in the outer retina. (**B**): The boxed area in (**A)** as assessed by control stains with pre-immune serum (PIS) and secondary antibody only (2°) and stains for the blood vessels with isolectin B4 (red) and albumin (green). Nuclei were stained with DAPI. The magnification of the cyan and green boxed regions in the latter two are also shown. The retinal layer labeling is as described in Figure 1 and Figure 2. Scale bars = 25 µm.

Imaging by FA revealed an important general difference between hamsters and mice. The margins of the retinal capillaries were more diffused in the outer retina of hamsters (independent of structural abnormalities) than in mice, and in some regions of hamster retina, bright fluorescent spots were even present (Figure 2 and Figure 4). The intensity of these bright spots was dependent on the FA phase, an indicator of the blood vessel permeability. Accordingly, imaging by FA suggested that the blood vessel permeability is increased in hamster retina as compared to mouse retina. To follow up on this finding, hamster retina was stained for albumin, which normally remains restricted within blood vessels [41], and retinal blood vessels were delineated with isolectin B4, which binds to the vascular endothelium [42]. Albumin extravasation from some but not all retinal blood vessels was detected in hamster retina and correlated with bright fluorescent spots observed on FA (Figure 5). Thus, indeed, the permeability of some of the retinal blood vessels seems to be increased in hamsters.

### 3.5. Blood Glucose and Glycemic Control

**Figure 6:**
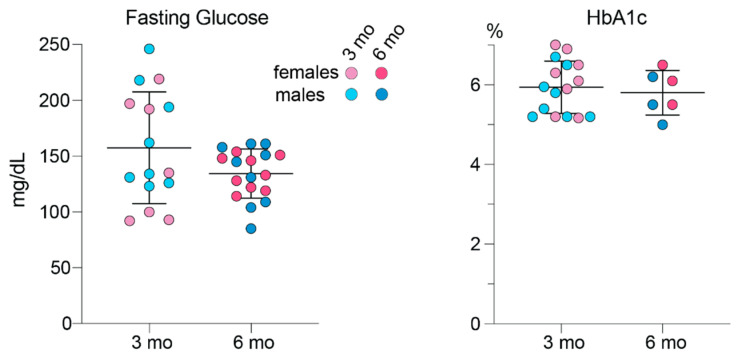
Blood glucose and glycemic control (HbA1C) in hamsters. The measurements were taken in individual animals: 3-month-old (3 mo) 7 females and 8 males for fasting glucose and 8 females and 8 males for hemoglobin A1c (HbA1c); and 6-month-old (6 mo) 9 females and 9 males for fasting glucose and 3 females and 3 males for HbA1c. Data represent the mean ± SD. No sex-based differences were detected, hence data from female and male animals were combined. No age differences were detected either.

Leakage of plasma from small intraretinal blood vessels into the interstitial space of the surrounding retina is a manifestation of early stage diabetic retinopathy [43]. Furthermore, Chinese hamsters are known to spontaneously develop diabetes [44,45]. Hence, we measured the hamster fasting blood glucose and HbA1c content, the latter being an indicator of long-term blood sugar control. At both 3 and 6 months of age, the levels of blood glucose and HbA1c were not significantly different between female and male hamsters and between the age groups (Figure 6). Notably, young hamsters had a higher interindividual variability in their fasting blood glucose levels with the ranges in females and males being 92–219 mg/dL and 123–246 mg/dL, respectively. The ranges in mature animals were smaller: 114–155 mg/dL in females and 85–161 mg/dL in males. Nevertheless, hamster variability in the blood HbA1c levels was not as high, thus suggesting that the blood glucose variability in young hamsters could possibly be due to the physiological stress caused by sedation. Overall, the mean blood glucose values in mature (6-month-old) hamsters (135 mg/dL in females and 134 mg/dL in males) were close to some of the reported values in the literature for Syrian hamsters (82 and 134 mg/dL in females; 72 and 120 mg/dL in males) [1,46]. Yet the mean HbA1c values (6.0 HbA1c% in females and 5.6 HbA1c% in males) were higher than those determined in non-diabetic Chinese hamsters of the same age but unspecified sex (~5 HbA1c%) [44].

### 3.6. Serum Lipids

**Figure 7:**
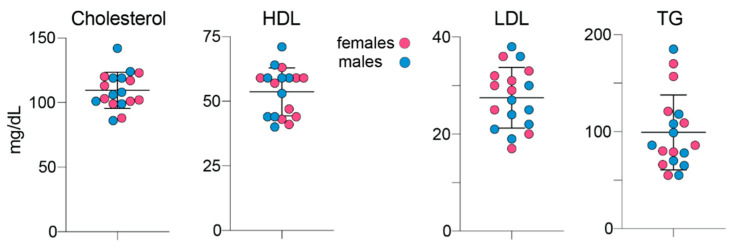
Fasting serum lipids in 6-month old hamsters. Data represent the mean ± SD of measurements in each hamster: 9 females and 9 males. No sex-based differences were detected, hence data from female and male animals were combined. HDL, high density lipoprotein cholesterol; LDL, low density lipoprotein cholesterol; TG, triglycerides.

The measurements were in mature hamsters and included those of total cholesterol, HDL and LDL cholesterol and triglycerides (Figure 7). No statistically significant differences were found between female and male hamsters in the levels of any of the measured serum lipids with the mean values, when both sexes were combined, being as follows: 109 mg/dL (serum cholesterol); 54 md/dL (HDL cholesterol); 28 mg/dL (LDL cholesterol); 100 mg/dL (triglycerides). Thus, the serum LDL to HDL cholesterol ratio in hamsters is approximately 1 to 2, which is indeed much higher than that measured previously by us in mice (approximately 1 to 17.5 or ~ 4 mg/dL to 70 mg/dL) [30].

### 3.7. Retinal Sterol Profiling

**Figure 8:**
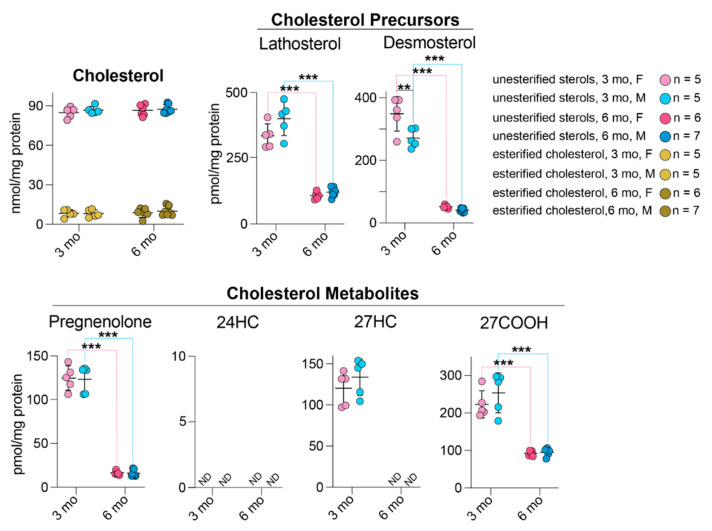
Retinal sterols in hamsters. Female (F) and male (M) animals were 3- and 6-months old (mo). Data represent the mean ± SD of the measurements in one retina in each hamster; *n* = number of hamsters. Statistical significance was assessed by one-way ANOVA with Tukey’s multiple comparisons test. No sex-based differences (except for desmosterol levels) were detected. **, *p* ≤ 0.01 ***, *p* ≤ 0.001. 24HC, 24-hydroxycholesterol; 27HC, 27-hydroxycholesterol; 27COOH, 5-cholestenoic acid; F, females; M, males; ND, not determined (the limit of detection is 1 pmol/mg protein).

Young and mature hamsters of both sexes were used and compared for the levels of seven sterols that were previously detected either in mouse or human retina [32]: cholesterol (unesterified and total), two cholesterol precursors (lathosterol and desmosterol), and four potential cholesterol metabolites (pregnenolone, 24-hydroxycholesterol (24HC), 27-hydroxycholesterol (27HC) and 5-cholestenoic acid (27COOH) (Figure 8). Lathosterol and desmosterol are the markers of cholesterol biosynthesis in neurons and astrocytes [47], respectively, and lathosterol is also a marker of the overall cholesterol biosynthesis in the retina [48]. Pregnenolone and 24HC are the products of activities of retinal cholesterol-metabolizing enzymes CYP11A1 and CYP46A, respectively, whereas 27HC and 27COOH are the metabolites of the sequential hydroxylation reactions catalyzed by CYP27A1 [49].

No sex-based differences were found in the levels of any sterols (except desmosterol) in all groups of tested hamsters. Moreover, sterol levels did not seem to be affected by retinal structural abnormalities. The content of cholesterol (total, unesterified, and esterified) did not vary with age. Conversely, the content of all other sterols showed age-dependent changes, which suggested that the retinal cholesterol turnover is much slower in mature than in young animals. Indeed, the retinal content of cholesterol precursors lathosterol and desmosterol was 3.3- and 6.9-fold lower, in 6-month-old hamsters than 3-month-old animals, respectively. Similarly, the content of cholesterol metabolites pregnenolone and 27COOH was also lower at 6 months of age than 3 months (8.3-fold and 2.5-fold, respectively), and 27HC was not even detectable in mature hamsters. Of the other cholesterol metabolites, 24HC was not detectable at any age; apparently, in both mature and young animals, CYP46A1 plays a minor role in retinal cholesterol elimination, and it is mostly CYP27A1 and CYP11A1 that eliminate cholesterol from hamster retina.

To gain further insights into retinal cholesterol maintenance in hamsters, we compared the quantifications in the present work with those in our previous studies that characterized mouse and human retinas [32,50,51] (Table 2). This comparative analysis will be presented in the Discussion section.

### 3.8. Retinal Cholesterol Distribution

**Figure 9:**
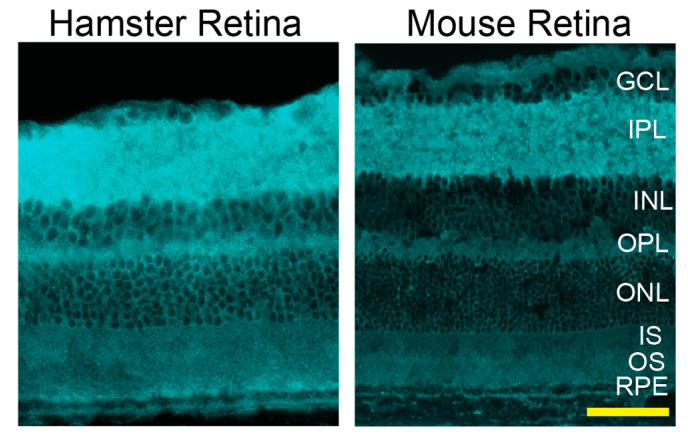
Retinal distribution of unesterified cholesterol in hamsters and mice (C57BL/6J). A fluorescent antibiotic filipin was used for staining of retinal cross sections (cyan). Both images are representative (3 females and 2 males for hamsters and 4 females and 2 males for mice). The retinal layer labeling is as described in Figure 1 and Figure 2. Scale bar = 50 µm.

Filipin, a validated histochemistry agent for staining of cholesterol [52], was used. Cholesterol was found in every retinal layer, and its distribution in hamster retina was similar to that in mouse retina (Figure 9).

## 4. Discussion

The present study provided several important findings about Syrian hamsters as an animal model and a model for studies of retinal cholesterol homeostasis. Indeed, sterol profiling of hamster retina revealed that its steady state cholesterol content was much higher than that found in mouse and human retinas (87, 35, and 49 nmol/mg of total retina protein, respectively, Table 2), which is apparently a peculiarity of hamster retina. The reason for such high cholesterol content is currently unknown.

The measurements of cholesterol precursors lathosterol and desmosterol and their ratios to cholesterol were lower in mature hamster retina than mature mouse retina, thus indicating that the rate of local cholesterol biosynthesis in hamster retina could be lower than that in mouse retina. This lower rate could, in turn, reflect an increased cholesterol supply from the systemic circulation as the serum lipid profiles are quite different in the two species: the serum LDL and LDL to HDL ratio is much higher in hamsters (28 mg/dL and 1:2, respectively, Figure 7) than in mice (4 mg/dL and 1:17.5, respectively) [30]. Accordingly, there are much more apolipoprotein particles in hamster (and human) than in mouse serum, which deliver cholesterol to different tissues, including the retina. The present work provides support for our next study, in which we will quantify retinal cholesterol input in hamsters: local biosynthesis and cholesterol uptake from the systemic circulation. The results will be compared with those obtained previously in mice [22] and will quantitatively ascertain whether hamsters are a better model than mice of retinal cholesterol input.

With respect to retinal cholesterol elimination, 27COOH, the product of CYP27A1 activities, was the most abundant cholesterol metabolite in hamsters and humans but not in mice (Table 2). Additionally, the pregnenolone to cholesterol and 27COOH to cholesterol ratios were more similar between hamsters and humans than mice and humans, which is further evidence in support of hamsters as a better model for retinal cholesterol homeostasis than mice. 24OH, the product of CYP46A1 activities, was not detectable in hamsters, and the 24OH to cholesterol ratio was extremely low in humans (0.05). In contrast, this ratio was much higher in mice (0.2).

Unexpectedly, we documented that ~23% of hamsters had two types of retinal structural abnormalities (Figure 4), both of which were similar to the manifestations of Rd8 in mice [40]. Yet, we have not detected the Rd8-causing mutation in hamsters. Thus, it is possible that in the hamster *Crb1*, there could be a mutation outside of exon 9, which like the Rd8-causing mutation, truncates the transmembrane and cytoplasmic domains of CRB1 and leads to the Rd8-like manifestations. Alternatively, a number of the *Crb1* splice variants have been reported [40,53], and many of them lack the transmembrane and cytoplasmic domains of CRB1. Accordingly, it is possible that these splice variants could lead to retinal structural abnormalities as well. Another consideration is that the phenotypic characteristics of the *Crb1* mutations could be due to environmental and genetic modifiers, and *Crb1* could be a susceptibility locus whose mutant forms interact with the mutations at other loci for the structural abnormality to develop [40]. Sequencing of the full hamster *Crb1* and possibly hamster genome is necessary to clarify a genetic reason for the observed structural abnormalities. In the meantime, the unknown etiology of these abnormalities suggests that prior to experiments, all hamsters should be screened by SD-OCT and then, depending on the goal of the study, a decision should be made whether to exclude hamsters with structural abnormalities to avoid the possibility of confounding contributions.

In addition to structural abnormalities, the permeability of the retinal blood vessels seemed to be increased in hamsters as compared with that in mice (Figure 2 and Figure 4). The HbA1c levels appeared to be elevated in 3- (6%) and 6-month-old (5.6%) hamsters (Figure 6) but we did not investigate whether there was a link between the hamster blood glycemic control and permeability of the retinal blood vessels. This is certainly a possibility as in the *Cricetinae* subfamily that includes hamsters, both Chinese and South African hamsters are known as models of spontaneous, non-insulin-dependent diabetes mellitus. The disease develops at 2–6 or 6–16 weeks of age, depending on the line, and ranges from mild (blood glucose: 150–250 mg/dL; HbA1c: 5–7%) to severe (blood glucose: up to 500 mg/dL; HbA1c: up to 22%) [1,44,45,54]. Herein, we characterized Syrian hamsters up to 6 months (or 26 weeks) of age, and their blood glucose was below 150 mg/dL and the HbA1c levels were no higher than 5.6% at 6 months of age (Figure 6). Perhaps Syrian hamsters at 3–6 months of age represent a model of prediabetes with signs of early stage diabetic retinopathy. The latter is unusual because in humans increased retinal vasopermeability typically appears as a complication of diabetes and frequently occurs in individuals with poor glycemic control [43]. Obviously, studies of Syrian hamsters older than 6 months are required to ascertain whether their blood glucose, glycemic control, and the permeability of their retinal blood vessels are linked and increase with age.

When comparing mice and hamsters as retinal models, one needs to keep in mind that the retina of both species lacks a macula, which is present in humans and densely packed with photoreceptor cells, especially cones, allowing for sharp, clear, and colored vision [55,56]. Hamster, but not mouse, retina has the area centralis, a specialized region that has some features of the human macula (e.g., increased density of ganglion cells [36]). In addition, hamster retina is larger than mouse retina (Table 1), and individual hamster retinas can be used for quantifications of cholesterol and other retinal sterols (Table 2). This is in contrast to mice, whose retinas need to be combined from 3–4 eyes to quantify pregnenolone, 24HC, 27HC, and 27COOH. Plus, as our study suggests, retinal cholesterol input could be affected by whole-body cholesterol maintenance, which is more similar in hamsters and humans than mice and humans, as is retinal cholesterol output.

A limitation of hamsters as an animal model is that they become mature 3 months later than mice and are more difficult to handle and breed than mice. In addition, adjustments must be made to the equipment used for in vivo retinal imaging as animal versions of these instruments are mostly designed for mice and rats. Importantly, studies of hamsters require a lot of documentation and presence of a veterinarian when new types of experiments are conducted as hamsters are a protected species by the United States Department of Agriculture.

Our study has several limitations, which include a lack of evaluations of aging hamsters (older than 1.5 years old), unclarified etiology of retinal structural abnormalities, and a lack of unambiguous confirmation of the area centralis location.

In summary, we used young and mature hamsters and conducted their ocular biometric assessments, imaged their retina in vivo, studied hamster retina by histo- and immunohistochemistry, genotyped for the mutations affecting retinal structure and measured their blood chemistry and retinal sterols. This comprehensive characterization provided a basis for subsequent studies of hamster retina as a model for retinal cholesterol homeostasis and therapeutic interventions aimed at lowering retinal cholesterol.

## 5. Patents

This study did not lead to any patents.

## Institutional Animal Care and Use Committee

All animal procedures were approved by the Case Western Reserve University (Cleveland, OH, USA) Institutional Animal Care and Use Committee (protocol 2020-0029, approved on 23 July 2020) and conformed to the guidelines of the United States Department of Agriculture, the American Veterinary Association Panel on Euthanasia, and the Association for Research in Vision and Ophthalmology.

## Figures and Tables

**Table 1:** Ocular biometric assessments in Syrian hamsters and C57BL/6J mice. Data represent the mean ± SD. Numbers in parentheses indicate the number of assessed animals (F, female; M, male). Only one eye from each animal was used for measurements of the axial eye length; data from both eyes were averaged for measurements of total retinal thickness. All data were analyzed by a two-tailed, unpaired Student’s *t*-test, except those for total retinal thickness, which were assessed by an unpaired non-parametric Mann–Whitney test.

Biometric Assessment	Hamsters		Mice	
	3-Month-Old	6-Month-Old	3-Month-Old	6-Month-Old
Axial eye length by ruler (mm)	5.9 ± 0.1 ^a^ (3F + 3M)	6.0 ± 0.0 ^a^ (3F + 3M)	3.2 ± 0.1 (4F + 3M)	3.5 ± 0.3 (4F + 3M)
Axial eye length by SD-OCT (mm)	6.0 ± 0.1 (1F + 2M)	6.1 ± 0.1 (3F + 3M)	ND	ND
Retina wet weight (mg)	6.9 ± 0.5 ^a^ (5F + 5M)	7.7 ± 0.9 ^a^ (7F + 6M)	2.3 ± 0.1 (9F +10M)	2.4 ± 0.1 (5F + 7M)
Total thickness of the retinal cross section by SD-OCT (µm)	204.2 ± 9.7 ^a^ (5F + 6M)	200.7 ± 3.7 ^a^ (6F + 4M)	229.4 ± 8.8 (5F + 7M)	237.6 ± 15.0 (4F + 4M)

^a^ Statistically significant difference (*p* < 0.01) as compared to mice of the same age. No statistically significant differences were detected between 3- and 6-month-old female and male animals of the same species. ND, not determined.

**Table 2:** Retinal amounts of free sterols and their relative ratios to cholesterol in different species. Numbers in parenthesis indicate the number of assessed animals or humans (F, female; M, male).

Retinal Sterol or Sterol/Cholesterol Ratio	Hamsters, 3-mo (5F + 5M)	Hamsters, 6-mo (5F + 5M)	Mice * 6-mo (6F + 6M)	Humans ** (3M)	Change Factor Relevant to Humans: 6-mo Hamsters; 6-mo Mice
Cholesterol, nmol/mg protein	86 ± 3	87 ± 4	35 ± 4	49 ± 7	1.78; 0.71
Lathosterol, pmol/mg protein	367 ± 62	115 ± 17	85 ± 6	NM	NA
Desmosterol, pmol/mg protein	310 ± 58	45± 8	54 ± 4	NM	NA
Pregnenolone, pmol/mg protein	124 ± 38	15 ± 3	25 ± 6	2.7 ± 0.8	5.55; 9.26
27HC, pmol/mg protein	127 ± 21	ND	ND	ND	NA
27COOH, pmol/mg protein	238 ± 46	94 ± 9	7.7 ± 0.9	79 ± 44	1.19; 0.10
24HC, pmol/mg protein	ND	ND	6.1 ± 1.0	2.4 ± 1.7	NA; 2.54
Lathosterol/cholesterol, pmol/nmol	4.3	1.3	2.5	NM	NA
Desmosterol/cholesterol, pmol/nmol	3.6	0.5	1.6	NM	NA
Pregnenolone/cholesterol pmol/nmol	1.4	0.17	0.84	0.06	2.83; 14
27HC/cholesterol, pmol/nmol	1.5	ND	ND	ND	NA
27COOH/cholesterol, pmol/nmol	2.8	1.1	0.22	1.6	0.69; 0.14
24HC/cholesterol, pmol/nmol	ND	ND	0.20	0.05	NA; 4.0

* Data are taken from [50,51] and represent measurements of cholesterol, lathosterol, desmosterol, and 24-hydroxycholesterol (24HC) in individual retinas of C57BL/6J mice. For the 27-hydroxycholesterol (27HC) and 5-cholestenoic acid (27COOH) quantifications, pooled samples of both retinas from 3 to 4 mice were used. ** Data are taken from [32] and represent measurements in individual retinas. mo, month old; NA, not applicable; NM, not measured; ND, not detectable, the limit of detection is 1 pmol/mg protein.

## Data Availability

Data are contained within the article or Supplementary Material.

## Supplementary Materials

The following are available online at https://www.mdpi.com/article/10.3390/biology10101003/s1. This article contains Table S1 (A summary of hamster characterizations by animal); Figure S1 (Sequence alignment of hamster and mouse *Crb1* exon 9 involved in retinal degeneration 8, and hamster genotyping in this region), and Figure S2 (Sequence alignment of hamster and mouse Pde6β exon 7 involved in retinal degeneration 1 and hamster genotyping in this region).

## Abbreviations

24HC: 24-hydroxycholesterol; 27COOH, 5-cholestenoic acid; 27HC, 27-hydroxycholesterol; F, female; HbA1c, hemoglobin A1c; HDL, high density lipoprotein; H&E, hematoxylin and eosin; FA, fluorescein angiography; FI, fundus imaging by fundoscope; LDL, low density lipoprotein; M, male; NGS, normal goat serum; Rd1, retinal degeneration 1; Rd8, retinal degeneration 8; SD-OCT, spectral-domain optical coherence tomography; PBS, phosphate-buffered saline; XMV, xenotropic murine leukemia virus 28.
