# Supplementary material for: Characterizations of Hamster Retina as a Model for Studies of Retinal Cholesterol Homeostasis
Source: Biology (Basel). 2021 Oct 6;10(10):1003. doi: 10.3390/biology10101003 (PMC8533155; doi:10.3390/biology10101003)
Supplement: Supplementary file 1 [file biology-10-01003-s001.zip › biology-1401551-supplementary.pdf]

Characterizations of Hamster Retina as a Model For Studies of Retinal Cholesterol Homeostasis

Nicole El-Darzi[1], Natalia Mast[1], Brian Dailey[1], John Denker[1], Yong Li[1], Joseph Vance [2], and Irina A. Pikuleva[1]

[1]Department of Ophthalmology and Visual Sciences, Case Western Reserve University, Cleveland, Ohio

[2]Spective LLC, Durham, North Carolina

Table S1. A summary of hamster characterizations by animal. The superscript near the hamster designation indicates retinal lesion on SD-OCT and the affected eye (OD or OS): w, occasional waviness of the ellipsoid zone of the photoreceptors; pr, the cone-like protrusions from the inner to the outer retina. F, females; M, males.

Hamster$^{leison, eye}$	3-month old				6-month old				
	In vivo animal characterizations		*In vitro* retinal characterizations		*In vivo* animal characterizations			*In vitro* retinal characterizations	
	In vivo imaging	Blood glucose/ HbA1c	Sterols, OD/OS	Histology, OD/OS	*In vivo* imaging	Blood glucose/ HbA1c	Serum lipids	Sterols, OD/OS	Histology, OD/OS
F1	-	-/-	-/-	-/-	+	+/-	+	+/-	-/+
F2	-	-/-	-/-	-/-	+	+/-	+	+/-	-/-
F3$^{w, OS}$	-	-/-	-/-	-/-	+	+/-	+	-/-	+/+
F4	-	-/-	-/-	-/-	+	+/-	+	-/+	-/-
F5	-	-/-	-/-	-/-	+	+/-	+	+/-	-/+
F6	-	-/-	-/-	-/-	+	+/-	+	+/-	-/-
F7	+	+/+	-/-	-/-	+	+/+	+	+/-	-/+
F8	+	-/+	+/-	-/+	-	-/-	-	-/-	-/-
F9	+	+/+	-/-	-/-	+	+/+	+	-/+	+/-
F10$^{pr, OD\&OS}$	+	-/-	-/+	+/-	-	-/-	-	-/-	-/-
F11	+	+/+	-/-	-/-	+	+/+	+	+/-	-/+
F12	+	+/+	+/-	-/+	-	-/-	-	-/-	-/-
F13	+	+/+	-/+	+/-	-	-/-	-	-/-	-/-
F14$^{pr, OD\&OS}$	+	+/+	+/-	-/+	-	-/-	-	-/-	-/-
F15	+	+/+	-/+	+/-	-	-/-	-	-/-	-/-
M1$^{w, OS}$	-	-/-	-/-	-/-	+	+/-	+	-/-	+/+
M2$^{w, OS}$	-	-/-	-/-	-/-	+	+/-	+	+/-	-/+
M3	-	-/-	-/-	-/-	+	+/-	+	-/-	-/-
M4	-	-/-	-/-	-/-	+	+/-	+	+/-	-/-
M5	-	-/-	-/-	-/-	+	+/-	+	+/-	-/-
M6	-	-/-	-/-	-/-	+	+/-	+	-/+	+/-
M7	+	+/+	-/-	-/-	+	+/+	+	+/-	-/+
M8	+	+/-	-/-	-/-	+	+/+	+	+/-	-/+
M9	+	+/+	-/-	-/-	+	+/+	+	-/+	+/-
M10	+	-/+	+/-	-/+	-	-/-	-	-/-	-/-
M11	+	+/+	+/-	-/+	-	-/-	-	-/-	-/-
M12$^{w, OS}$	+	+/+	+/-	-/+	-	-/-	-	-/-	-/-
M13$^{pr, OD\&OS}$	+	+/+	-/+	-/+	-	-/-	-	-/-	-/-
M14	+	+/+	-/+	-/+	-	-/-	-	-/-	-/-
M15	+	+/+	+/-	-/+	-	-/-	-	-/-	-/-

```
Hamster    TTACTGGCTGCTTGCCACTAAATGCCTGCCACTCCAGCCAGTGTTTGCATGGAGGAAACTGTGAAGACAGCTACAGCTCTTATCAGTGTTCCTGTCTCTTG
           ---T--G--C--L--P--L--N--A--C--H--S--S--Q--C--L--H--G--G--N--C--E--D--S--Y--S--S--Y--Q--C--S--C--L--L-
F1         TTACTGGCTGCTTGCCACTAAATGCCTGCCACTCCAGCCAGTGTTTGCATGGAGGAAACTGTGAAGACAGCTACAGCTCTTATCAGTGTTCCTGTCTCTTG 101
F2         TTACTGGCTGCTTGCCACTAAATGCCTGCCACTCCAGCCAGTGTTTGCATGGAGGAAACTGTGAAGACAGCTACAGCTCTTATCAGTGTTCCTGTCTCTTG
F3         TTACTGGCTGCTTGCCACTAAATGCCTGCCACTCCAGCCAGTGTTTGCATGGAGGAAACTGTGAAGACAGCTACAGCTCTTATCAGTGTTCCTGTCTCTTG
F4         TTACTGGCTGCTTGCCACTAAATGCCTGCCACTCCAGCCAGTGTTTGCATGGAGGAAACTGTGAAGACAGCTACAGCTCTTATCAGTGTTCCTGTCTCTTG
F5         TTACTGGCTGCTTGCCACTAAATGCCTGCCACTCCAGCCAGTGTTTGCATGGAGGAAACTGTGAAGACAGCTACAGCTCTTATCAGTGTTCCTGTCTCTTG
F6         TTACTGGCTGCTTGCCACTAAATGCCTGCCACTCCAGCCAGTGTTTGCATGGAGGAAACTGTGAAGACAGCTACAGCTCTTATCAGTGTTCCTGTCTCTTG
F7         TTACTGGCTGCTTGCCACTAAATGCCTGCCACTCCAGCCAGTGTTTGCATGGAGGAAACTGTGAAGACAGCTACAGCTCTTATCAGTGTTCCTGTCTCTTG
F8         TTACTGGCTGCTTGCCACTAAATGCCTGCCACTCCAGCCAGTGTTTGCATGGAGGAAACTGTGAAGACAGCTACAGCTCTTATCAGTGTTCCTGTCTCTTG
F9         TTACTGGCTGCTTGCCACTAAATGCCTGCCACTCCAGCCAGTGTTTGCATGGAGGAAACTGTGAAGACAGCTACAGCTCTTATCAGTGTTCCTGTCTCTTG
F10        TTACTGGCTGCTTGCCACTAAATGCCTGCCACTCCAGCCAGTGTTTGCATGGAGGAAACTGTGAAGACAGCTACAGCTCTTATCAGTGTTCCTGTCTCTTG
F11        TTACTGGCTGCTTGCCACTAAATGCCTGCCACTCCAGCCAGTGTTTGCATGGAGGAAACTGTGAAGACAGCTACAGCTCTTATCAGTGTTCCTGTCTCTTG
F12        TTACTGGCTGCTTGCCACTAAATGCCTGCCACTCCAGCCAGTGTTTGCATGGAGGAAACTGTGAAGACAGCTACAGCTCTTATCAGTGTTCCTGTCTCTTG
F13        TTACTGGCTGCTTGCCACTAAATGCCTGCCACTCCAGCCAGTGTTTGCATGGAGGAAACTGTGAAGACAGCTACAGCTCTTATCAGTGTTCCTGTCTCTTG
F14        TTACTGGCTGCTTGCCACTAAATGCCTGCCACTCCAGCCAGTGTTTGCATGGAGGAAACTGTGAAGACAGCTACAGCTCTTATCAGTGTTCCTGTCTCTTG
F15        TTACTGGCTGCTTGCCACTAAATGCCTGCCACTCCAGCCAGTGTTTGCATGGAGGAAACTGTGAAGACAGCTACAGCTCTTATCAGTGTTCCTGTCTCTTG
M1         TTACTGGCTGCTTGCCACTAAATGCCTGCCACTCCAGCCAGTGTTTGCATGGAGGAAACTGTGAAGACAGCTACAGCTCTTATCAGTGTTCCTGTCTCTTG
M2         TTACTGGCTGCTTGCCACTAAATGCCTGCCACTCCAGCCAGTGTTTGCATGGAGGAAACTGTGAAGACAGCTACAGCTCTTATCAGTGTTCCTGTCTCTTG
M3         TTACTGGCTGCTTGCCACTAAATGCCTGCCACTCCAGCCAGTGTTTGCATGGAGGAAACTGTGAAGACAGCTACAGCTCTTATCAGTGTTCCTGTCTCTTG
M4         TTACTGGCTGCTTGCCACTAAATGCCTGCCACTCCAGCCAGTGTTTGCATGGAGGAAACTGTGAAGACAGCTACAGCTCTTATCAGTGTTCCTGTCTCTTG
M5         TTACTGGCTGCTTGCCACTAAATGCCTGCCACTCCAGCCAGTGTTTGCATGGAGGAAACTGTGAAGACAGCTACAGCTCTTATCAGTGTTCCTGTCTCTTG
M6         TTACTGGCTGCTTGCCACTAAATGCCTGCCACTCCAGCCAGTGTTTGCATGGAGGAAACTGTGAAGACAGCTACAGCTCTTATCAGTGTTCCTGTCTCTTG
M7         TTACTGGCTGCTTGCCACTAAATGCCTGCCACTCCAGCCAGTGTTTGCATGGAGGAAACTGTGAAGACAGCTACAGCTCTTATCAGTGTTCCTGTCTCTTG
M8         TTACTGGCTGCTTGCCACTAAATGCCTGCCACTCCAGCCAGTGTTTGCATGGAGGAAACTGTGAAGACAGCTACAGCTCTTATCAGTGTTCCTGTCTCTTG
M9         TTACTGGCTGCTTGCCACTAAATGCCTGCCACTCCAGCCAGTGTTTGCATGGAGGAAACTGTGAAGACAGCTACAGCTCTTATCAGTGTTCCTGTCTCTTG
M10        TTACTGGCTGCTTGCCACTAAATGCCTGCCACTCCAGCCAGTGTTTGCATGGAGGAAACTGTGAAGACAGCTACAGCTCTTATCAGTGTTCCTGTCTCTTG
M11        TTACTGGCTGCTTGCCACTAAATGCCTGCCACTCCAGCCAGTGTTTGCATGGAGGAAACTGTGAAGACAGCTACAGCTCTTATCAGTGTTCCTGTCTCTTG
M12        TTACTGGCTGCTTGCCACTAAATGCCTGCCACTCCAGCCAGTGTTTGCATGGAGGAAACTGTGAAGACAGCTACAGCTCTTATCAGTGTTCCTGTCTCTTG
M13        TTACTGGCTGCTTGCCACTAAATGCCTGCCACTCCAGCCAGTGTTTGCATGGAGGAAACTGTGAAGACAGCTACAGCTCTTATCAGTGTTCCTGTCTCTTG
M14        TTACTGGCTGCTTGCCACTAAATGCCTGCCACTCCAGCCAGTGTTTGCATGGAGGAAACTGTGAAGACAGCTACAGCTCTTATCAGTGTTCCTGTCTCTTG
M15        TTACTGGCTGCTTGCCACTAAATGCCTGCCACTCCAGCCAGTGTTTGCATGGAGGAAACTGTGAAGACAGCTACAGCTCTTATCAGTGTTCCTGTCTCTTG

Mouse      TTACTGGCTGTTTGCCATCAAATGCCTGCCACTCCAGCCCCTGTTTGCATGGAGGAAACTGTGAAGACAGCTACAGTTCTTATCGGTGTGCCTGTCTCTCG
Rd8 mut    TTACTGGCTGTTTGCCATCAAATGCCTGCCACTCCAGCCCCTGTTTGCATGGAGGAAACTGTGAAGACAGCTACAGTTCTTAT-GGTGTGCCTGTCTCTCG
           ********** ******  ********************  *********************************** ******  **** ********* *
```

```
Hamster   GGATGGTCGGGGACACACTGTGAAATCAACATTGATGAATGCGGTTCTAACCCCTGTATCCATGGCAACTGCTCTGATGGAGTTGCAGCCTACCACT
          -G--W--S--G--T--H--C--E--I--N--I--D--E--C--G--S--N--P--C--I--H--G--N--C--S--D--G--V--A--A--Y--H--
F1        GGATGGTCGGGGACACACTGTGAAATCAACATTGATGAATGCGGTTCTAACCCCTGTATCCATGGCAACTGCTCTGATGGAGTTGCAGCCTACCACT 198
F2        GGATGGTCGGGGACACACTGTGAAATCAACATTGATGAATGCGGTTCTAACCCCTGTATCCATGGCAACTGCTCTGATGGAGTTGCAGCCTACCACT
F3        GGATGGTCGGGGACACACTGTGAAATCAACATTGATGAATGCGGTTCTAACCCCTGTATCCATGGCAACTGCTCTGATGGAGTTGCAGCCTACCACT
F4        GGATGGTCGGGGACACACTGTGAAATCAACATTGATGAATGCGGTTCTAACCCCTGTATCCATGGCAACTGCTCTGATGGAGTTGCAGCCTACCACT
F5        GGATGGTCGGGGACACACTGTGAAATCAACATTGATGAATGCGGTTCTAACCCCTGTATCCATGGCAACTGCTCTGATGGAGTTGCAGCCTACCACT
F6        GGATGGTCGGGGACACACTGTGAAATCAACATTGATGAATGCGGTTCTAACCCCTGTATCCATGGCAACTGCTCTGATGGAGTTGCAGCCTACCACT
F7        GGATGGTCGGGGACACACTGTGAAATCAACATTGATGAATGCGGTTCTAACCCCTGTATCCATGGCAACTGCTCTGATGGAGTTGCAGCCTACCACT
F8        GGATGGTCGGGGACACACTGTGAAATCAACATTGATGAATGCGGTTCTAACCCCTGTATCCATGGCAACTGCTCTGATGGAGTTGCAGCCTACCACT
F9        GGATGGTCGGGGACACACTGTGAAATCAACATTGATGAATGCGGTTCTAACCCCTGTATCCATGGCAACTGCTCTGATGGAGTTGCAGCCTACCACT
F10       GGATGGTCGGGGACACACTGTGAAATCAACATTGATGAATGCGGTTCTAACCCCTGTATCCATGGCAACTGCTCTGATGGAGTTGCAGCCTACCACT
F11       GGATGGTCGGGGACACACTGTGAAATCAACATTGATGAATGCGGTTCTAACCCCTGTATCCATGGCAACTGCTCTGATGGAGTTGCAGCCTACCACT
F12       GGATGGTCGGGGACACACTGTGAAATCAACATTGATGAATGCGGTTCTAACCCCTGTATCCATGGCAACTGCTCTGATGGAGTTGCAGCCTACCACT
F13       GGATGGTCGGGGACACACTGTGAAATCAACATTGATGAATGCGGTTCTAACCCCTGTATCCATGGCAACTGCTCTGATGGAGTTGCAGCCTACCACT
F14       GGATGGTCGGGGACACACTGTGAAATCAACATTGATGAATGCGGTTCTAACCCCTGTATCCATGGCAACTGCTCTGATGGAGTTGCAGCCTACCACT
F15       GGATGGTCGGGGACACACTGTGAAATCAACATTGATGAATGCGGTTCTAACCCCTGTATCCATGGCAACTGCTCTGATGGAGTTGCAGCCTACCACT
M1        GGATGGTCGGGGACACACTGTGAAATCAACATTGATGAATGCGGTTCTAACCCCTGTATCCATGGCAACTGCTCTGATGGAGTTGCAGCCTACCACT
M2        GGATGGTCGGGGACACACTGTGAAATCAACATTGATGAATGCGGTTCTAACCCCTGTATCCATGGCAACTGCTCTGATGGAGTTGCAGCCTACCACT
M3        GGATGGTCGGGGACACACTGTGAAATCAACATTGATGAATGCGGTTCTAACCCCTGTATCCATGGCAACTGCTCTGATGGAGTTGCAGCCTACCACT
M4        GGATGGTCGGGGACACACTGTGAAATCAACATTGATGAATGCGGTTCTAACCCCTGTATCCATGGCAACTGCTCTGATGGAGTTGCAGCCTACCACT
M5        GGATGGTCGGGGACACACTGTGAAATCAACATTGATGAATGCGGTTCTAACCCCTGTATCCATGGCAACTGCTCTGATGGAGTTGCAGCCTACCACT
M6        GGATGGTCGGGGACACACTGTGAAATCAACATTGATGAATGCGGTTCTAACCCCTGTATCCATGGCAACTGCTCTGATGGAGTTGCAGCCTACCACT
M7        GGATGGTCGGGGACACACTGTGAAATCAACATTGATGAATGCGGTTCTAACCCCTGTATCCATGGCAACTGCTCTGATGGAGTTGCAGCCTACCACT
M8        GGATGGTCGGGGACACACTGTGAAATCAACATTGATGAATGCGGTTCTAACCCCTGTATCCATGGCAACTGCTCTGATGGAGTTGCAGCCTACCACT
M9        GGATGGTCGGGGACACACTGTGAAATCAACATTGATGAATGCGGTTCTAACCCCTGTATCCATGGCAACTGCTCTGATGGAGTTGCAGCCTACCACT
M10       GGATGGTCGGGGACACACTGTGAAATCAACATTGATGAATGCGGTTCTAACCCCTGTATCCATGGCAACTGCTCTGATGGAGTTGCAGCCTACCACT
M11       GGATGGTCGGGGACACACTGTGAAATCAACATTGATGAATGCGGTTCTAACCCCTGTATCCATGGCAACTGCTCTGATGGAGTTGCAGCCTACCAC
M12       GGATGGTCGGGGACACACTGTGAAATCAACATTGATGAATGCGGTTCTAACCCCTGTATCCATGGCAACTGCTCTGATGGAGTTGCAGCCTACCACT
M13       GGATGGTCGGGGACACACTGTGAAATCAACATTGATGAATGCGGTTCTAACCCCTGTATCCATGGCAACTGCTCTGATGGAGTTGCAGCCTACCACT
M14       GGATGGTCGGGGACACACTGTGAAATCAACATTGATGAATGCGGTTCTAACCCCTGTATCCATGGCAACTGCTCTGATGGAGTTGCAGCCTACCACT
M15       GGATGGTCGGGGACACACTGTGAAATCAACATTGATGAATGCGGTTCTAACCCCTGTATCCATGGCAACTGCTCTGATGGAGTTGCAGCCTACCACT

Mouse     GGATGGTCAGGGACACACTGTGAAATCAACATTGATGAGTGCTTTTCTAGCCCCTGTATCCATGGCAACTGCTCTGATGGAGTTGCAGCCTACCACT
Rd8 mut   GGATGGTCAGGGACACACTGTGAAATCAACATTGATGAGTGCTTTTCTAGCCCCTGTATCCATGGCAACTGCTCTGATGGAGTTGCAGCCTACCACT
          ******** ***************************** ***  ***** ***********************************************
```

Figure S1. Sequence alignment of hamster and mouse *Crb1* exon 9 involved in retinal degeneration 8, and hamster genotyping in this region. The DNA and amino acid sequences of the hamster protein are shown at the top. At the DNA level, the hamster to mice differences that lead to no change in amino acid are in blue, and those that change the amino acid are in red. At the protein level, conservative changes relative to mouse are in blue and mismatches are in red. The DNA sequences of wild type mice and mice carrying the retinal degeneration 8 mutation (Rd8 mut) are at the bottom. The mouse deletion is highlighted in green.

```
Hamster    CACACCCCCAGCTGATCACTGGGCCCTGGCCAGTGGCCTTCCAACCTATGTAGCAGAAAGTGGCTTT
           --T--P--P--A--D--H--W--A--L--A--S--G--L--P--T--Y--V--A--E--S--G--F-
F1         CACACCCCCAGCTGATCACTGGGCCCTGGCCAGTGGCCTTCCAACCTATGTAGCAGAAAGTGGCTTT  67
F2         CACACCCCCAGCTGATCACTGGGCCCTGGCCAGTGGCCTTCCAACCTATGTAGCAGAAAGTGGCTTT
F3         CACACCCCCAGCTGATCACTGGGCCCTGGCCAGTGGCCTTCCAACCTATGTAGCAGAAAGTGGCTTT
F4         CACACCCCCAGCTGATCACTGGGCCCTGGCCAGTGGCCTTCCAACCTATGTAGCAGAAAGTGGCTTT
F5         CACACCCCCAGCTGATCACTGGGCCCTGGCCAGTGGCCTTCCAACCTATGTAGCAGAAAGTGGCTTT
F6         CACACCCCCAGCTGATCACTGGGCCCTGGCCAGTGGCCTTCCAACCTATGTAGCAGAAAGTGGCTTT
F7         CACACCCCCAGCTGATCACTGGGCCCTGGCCAGTGGCCTTCCAACCTATGTAGCAGAAAGTGGCTTT
F8         CACACCCCCAGCTGATCACTGGGCCCTGGCCAGTGGCCTTCCAACCTATGTAGCAGAAAGTGGCTTT
F9         CACACCCCCAGCTGATCACTGGGCCCTGGCCAGTGGCCTTCCAACCTATGTAGCAGAAAGTGGCTTT
F10        CACACCCCCAGCTGATCACTGGGCCCTGGCCAGTGGCCTTCCAACCTATGTAGCAGAAAGTGGCTTT
F11        CACACCCCCAGCTGATCACTGGGCCCTGGCCAGTGGCCTTCCAACCTATGTAGCAGAAAGTGGCTTT
F12        CACACCCCCAGCTGATCACTGGGCCCTGGCCAGTGGCCTTCCAACCTATGTAGCAGAAAGTGGCTTT
F13        CACACCCCCAGCTGATCACTGGGCCCTGGCCAGTGGCCTTCCAACCTATGTAGCAGAAAGTGGCTTT
F14        CACACCCCCAGCTGATCACTGGGCCCTGGCCAGTGGCCTTCCAACCTATGTAGCAGAAAGTGGCTTT
F15        CACACCCCCAGCTGATCACTGGGCCCTGGCCAGTGGCCTTCCAACCTATGTAGCAGAAAGTGGCTTT
M1         CACACCCCCAGCTGATCACTGGGCCCTGGCCAGTGGCCTTCCAACCTATGTAGCAGAAAGTGGCTTT
M2         CACACCCCCAGCTGATCACTGGGCCCTGGCCAGTGGCCTTCCAACCTATGTAGCAGAAAGTGGCTTT
M3         CACACCCCCAGCTGATCACTGGGCCCTGGCCAGTGGCCTTCCAACCTATGTAGCAGAAAGTGGCTTT
M4         CACACCCCCAGCTGATCACTGGGCCCTGGCCAGTGGCCTTCCAACCTATGTAGCAGAAAGTGGCTTT
M5         CACACCCCCAGCTGATCACTGGGCCCTGGCCAGTGGCCTTCCAACCTATGTAGCAGAAAGTGGCTTT
M6         CACACCCCCAGCTGATCACTGGGCCCTGGCCAGTGGCCTTCCAACCTATGTAGCAGAAAGTGGCTTT
M7         CACACCCCCAGCTGATCACTGGGCCCTGGCCAGTGGCCTTCCAACCTATGTAGCAGAAAGTGGCTTT
M8         CACACCCCCAGCTGATCACTGGGCCCTGGCCAGTGGCCTTCCAACCTATGTAGCAGAAAGTGGCTTT
M9         CACACCCCCAGCTGATCACTGGGCCCTGGCCAGTGGCCTTCCAACCTATGTAGCAGAAAGTGGCTTT
M10        CACACCCCCAGCTGATCACTGGGCCCTGGCCAGTGGCCTTCCAACCTATGTAGCAGAAAGTGGCTTT
M11        CACACCCCCAGCTGATCACTGGGCCCTGGCCAGTGGCCTTCCAACCTATGTAGCAGAAAGTGGCTTT
M12        CACACCCCCAGCTGATCACTGGGCCCTGGCCAGTGGCCTTCCAACCTATGTAGCAGAAAGTGGCTTT
M13        CACACCCCCAGCTGATCACTGGGCCCTGGCCAGTGGCCTTCCAACCTATGTAGCAGAAAGTGGCTTT
M14        CACACCCCCAGCTGATCACTGGGCCCTGGCCAGTGGCCTTCCAACCTATGTAGCAGAAAGTGGCTTT
M15        CACACCCCCAGCTGATCACTGGGCCCTGGCCAGTGGCCTTCCAACCTATGTAGCAGAAAGTGGCTTT

Mouse      CACACCCCCGGCTGATCACTGGGCCCTGGCCAGTGGCCTTCCAACCTACGTAGCAGAAAGTGGCTTT
Rd1 mut    CACACCCCCGGCTGATCACTGGGCCCTGGCCAGTGGCCTTCCAACCTAAGTAGCAGAAAGTGGCTTT
           ********* ************************************** ******************
```

Figure S2. Sequence alignment of hamster and mouse *Pde6β* exon 7 involved in retinal degeneration 1, and hamster genotyping in this region. The DNA and amino acid sequences of the hamster protein are shown at the top. The DNA sequences of wild type mice and mice carrying the retinal degeneration 1 mutation (Rd1 mut) are at the bottom. The mouse mutation is highlighted in green. At the DNA level, the two hamster to mice differences (in blue) are silent and lead to no change in the amino acid.
